# Case Report: Hepatic inflammatory myofibroblastic tumor versus hepatic inflammatory pseudotumor in pediatric patients

**DOI:** 10.3389/fped.2026.1967031

**Published:** 2026-09-30

**Authors:** Jia-lin Tang, Miao-bing Wu, Xian-ping Jiang, Bin Wang, Zhi-yong Wang, Xiao-peng Ma, Jian-yao Wang

**Affiliations:** 1 Department of General Surgery, Shenzhen Children’s Hospital, Shenzhen, Guangdong, China; 2 Department of General Surgery, Shenzhen Pediatrics Institute of Shantou University Medical College, Shenzhen, Guangdong, China; 3 Department of Pathology, Shenzhen Children’s Hospital, Shenzhen, Guangdong, China

**Keywords:** case report, hepatic inflammatory myofibroblastic tumor, hepatic inflammatory pseudotumor, hepatic tumor, pediatric tumor

## Abstract

**Background:**

Hepatic Inflammatory Pseudotumor (HIPT) and Hepatic Inflammatory Myofibroblastic Tumor (HIMT) are rare pediatric liver lesions with highly similar clinical and pathological features, easily causing misdiagnosis. Accurate differentiation is critical as they require distinct clinical management strategies.

**Case summary:**

Two pediatric hepatic mass cases were included. An 8-year-old girl presented with chronic abdominal pain and was diagnosed with HIMT, with no evidence of recurrence at 12-month follow-up after surgical resection. A 12-year-old girl presented with recurrent fever and abdominal pain. Initial antibiotic treatment failed, and she underwent partial resection for biopsy followed by definitive surgery, along with postoperative antibiotics and low-dose prednisone therapy, and remained free of recurrence at 12-month follow-up.

**Conclusion:**

Based on the two cases in our center and narrative review, HIPT and HIMT differ in etiology, clinical manifestations, histopathology, immunohistochemistry and treatment regimens. Comprehensive analysis of all clinical information is required to improve diagnostic accuracy and reduce misdiagnosis and overtreatment of the two diseases.

## Introduction

The term “inflammatory pseudotumor” (IPT) was formally introduced by Umiker *et al*. in 1954 (1). In 1939, Brunn reported a pulmonary spindle cell lesion, retrospectively recognized as the earliest documented case of IPT. Historically classified as a benign, infection-driven reactive proliferation, IPT was later recognized to encompass lesions exhibiting aggressive features, including local invasion, recurrence, and rare metastasis. Subsequent immunohistochemical and molecular genetic analyses confirmed that a subset of lesions historically classified as IPT are true neoplasms composed of myofibroblasts, exhibiting low-grade or uncertain malignant potential (2). This evolving understanding culminated in a pivotal nosologic shift in 2002, when the World Health Organization (WHO) formally reclassified the subset as inflammatory myofibroblastic tumors (IMTs), fundamentally distinguishing them from non-neoplastic IPTs (3). Despite this nosologic distinction, historical literature frequently conflates the two entities. Owing to their overlapping clinical and radiologic features, IPT and IMT are commonly misdiagnosed in clinical practice. Importantly, their therapeutic strategies differ substantially, making precise differentiation critical. Consequently, a distinct characterization and synthesis of these two entities is necessary to minimize diagnostic errors and guide appropriate pediatric management.

Hepatic inflammatory pseudotumor (HIPT) is an exceptionally rare liver lesion, accounting for <1% of all hepatic masses. Unlike true neoplasms, HIPT is a non-neoplastic, reactive proliferation thought to arise from chronic inflammation (4–6). Patients typically present with nonspecific symptoms, most commonly dull abdominal pain and fever. Pathologically, it is a mass-forming lesion composed of fibroblasts or myofibroblasts admixed with inflammatory cells. Histologic examination reveals proliferative granulomatous components and fibrogenic stroma, which may occasionally undergo liquefactive necrosis.

Inflammatory myofibroblastic tumor (IMT) is a rare mesenchymal neoplasm characterized by a proliferation of spindle-shaped myofibroblasts interspersed with inflammatory cells. Although IMT can arise in multiple organs, hepatic involvement is rare, accounting for approximately 10% of all IMT cases (7, 8). Clinically, patients with hepatic IMT (HIMT) typically present with symptoms that closely mirror those of HIPT, including fever, weight loss, and constitutional symptoms (8). Over 60% of IMTs exhibit *anaplastic lymphoma kinase (ALK)* rearrangements, whereas ALK-negative cases may involve fusions of *ROS proto-oncogene 1, receptor tyrosine kinase* (*ROS1), neurotrophic tropomyosin receptor kinase (NTRK)*, or other kinases. These rearrangements confirm the neoplastic nature of IMT and provide actionable therapeutic targets, particularly in children (9, 10).

Herein, we describe one pediatric case each of HIPT and HIMT managed at our center. Additionally, we performed a narrative literature review of pediatric case reports identified through major electronic databases over the past 15 years to summarize their clinical characteristics. Our objective is to compare the potential differences between these two entities, thereby enhancing current clinical understanding and refining diagnostic and management approaches.

## Method

We conducted a narrative literature review covering the past 15 years, focusing on pediatric HIMT and HIPT. PubMed and Web of Science were systematically searched. References from selected articles were also screened manually. Both MeSH terms and free-text keywords were used. The primary search string was: (“inflammatory myofibroblastic tumor” OR “inflammatory pseudotumor”) AND (“liver” OR “hepatic”). After removing duplicates and irrelevant records, we extracted clinical variables from eligible case reports and case series. For variables with missing data, proportions were calculated based on the number of cases with available information; missing values were noted and excluded from the denominator. The final study population included 18 HIMT cases and 11 HIPT cases, including the two institutional index cases presented in this report.

### Clinical case

#### Case Ⅰ

An 8-year-old girl presented to our center with chronic abdominal pain. Initial imaging incidentally revealed a hepatic mass. She had a history of chronic sinusitis. Laboratory evaluation demonstrated an elevated C-reactive protein level (75.9 mg/L) and anemia (hemoglobin, 89 g/L); while comprehensive serologic and microbiologic testing yielded negative results. Abdominal ultrasonography identified a well-circumscribed hypoechoic mass measuring 5.5 × 4.2 × 4.9 cm in the left hepatic lobe, with imaging features that did not exclude malignancy (Figure 1A). Subsequent imaging with contrast-enhanced computed tomography (CT) and 3.0-T magnetic resonance imaging (MRI) showed a lesion measuring 4.9 × 4.7 × 3.9 cm in segment IV, which aligns with atypical focal nodular hyperplasia (Figures 2A,3A–E). Following preoperative preparation, the patient underwent abdominal surgical exploration. Intraoperatively, a grayish-white mass of moderate consistency was identified in the left hepatic lobe, predominantly involving segment IV (Figure 4A). Histopathological examination confirmed a HIMT (Figure 5). Immunohistochemical staining was positive for *ALK* and *smooth muscle actin (SMA)*, and negative for IgG4. Nonetheless, *ALK* break-apart FISH did not show any gene rearrangement. At 12-month follow-up, the patient remained asymptomatic with no clinical or radiographic evidence of recurrence.

**Figure 1:**
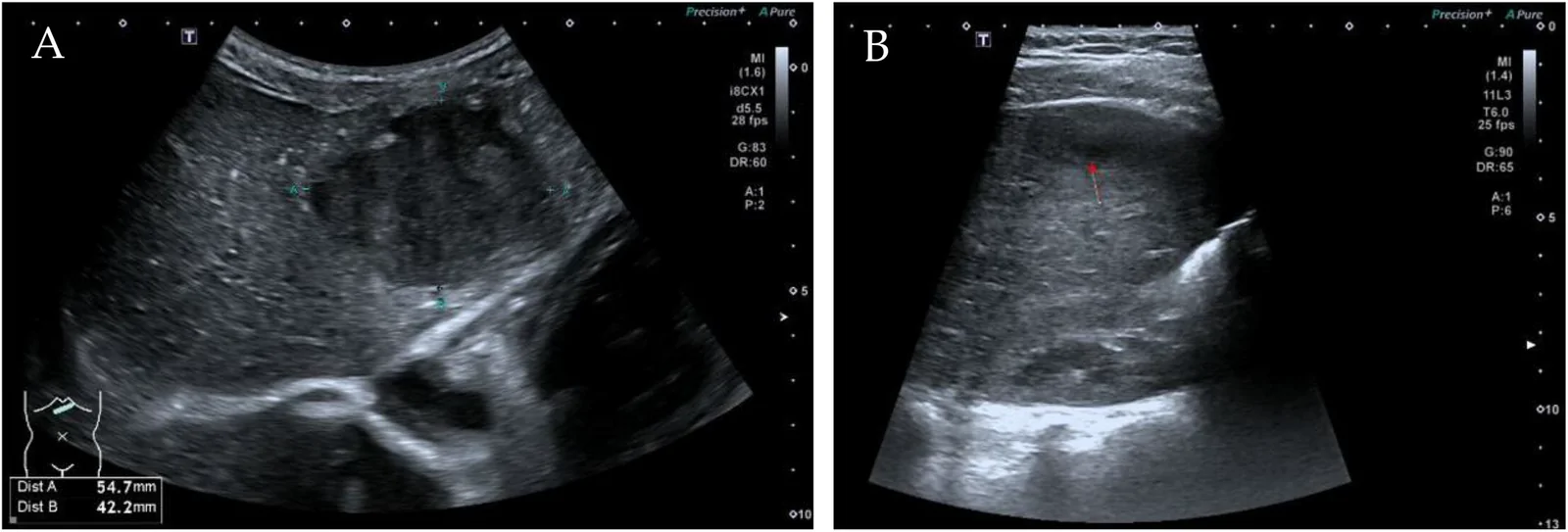
Ultrasonic imaging of HIMT and HIPT.

**Figure 2:**
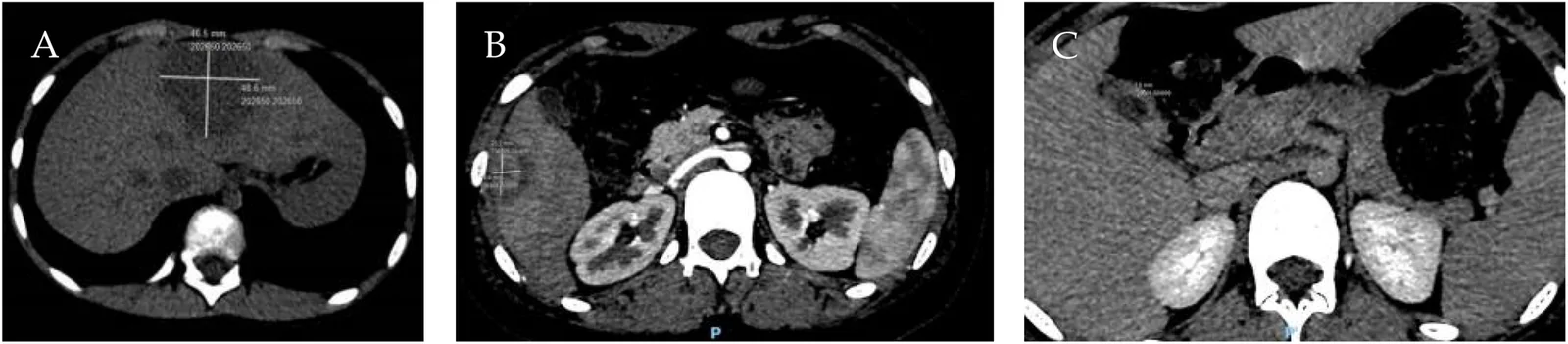
The computed tomography images of HIMT and HIPT.

**Figure 3:**
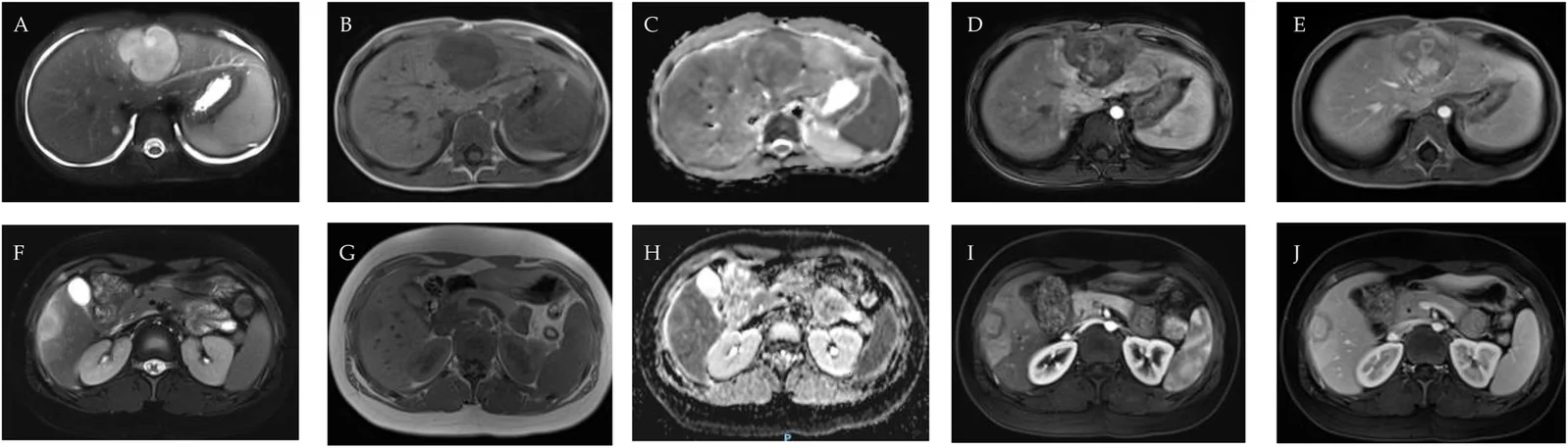
The magnetic resonance imaging of HIMT and HIPT.

**Figure 4:**
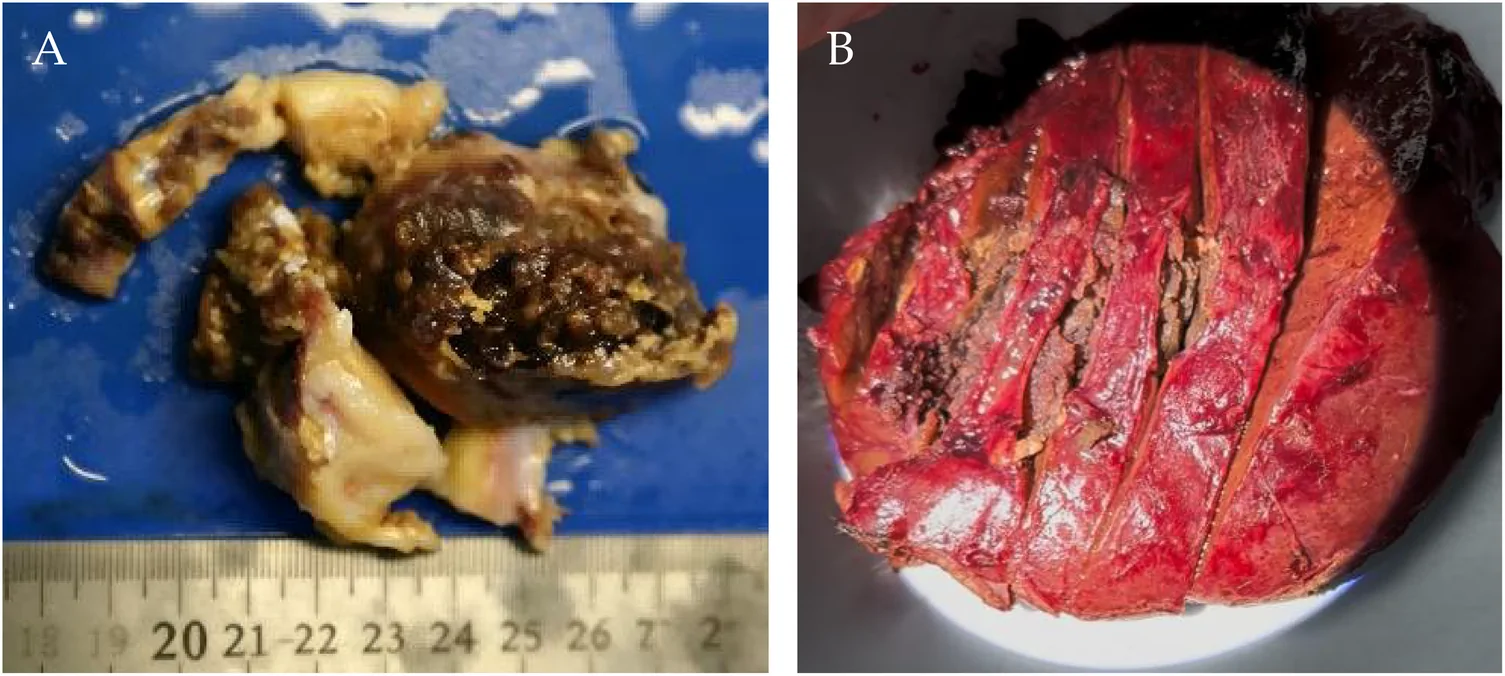
The images of HIMT and HIPT tumors.

**Figure 5:**
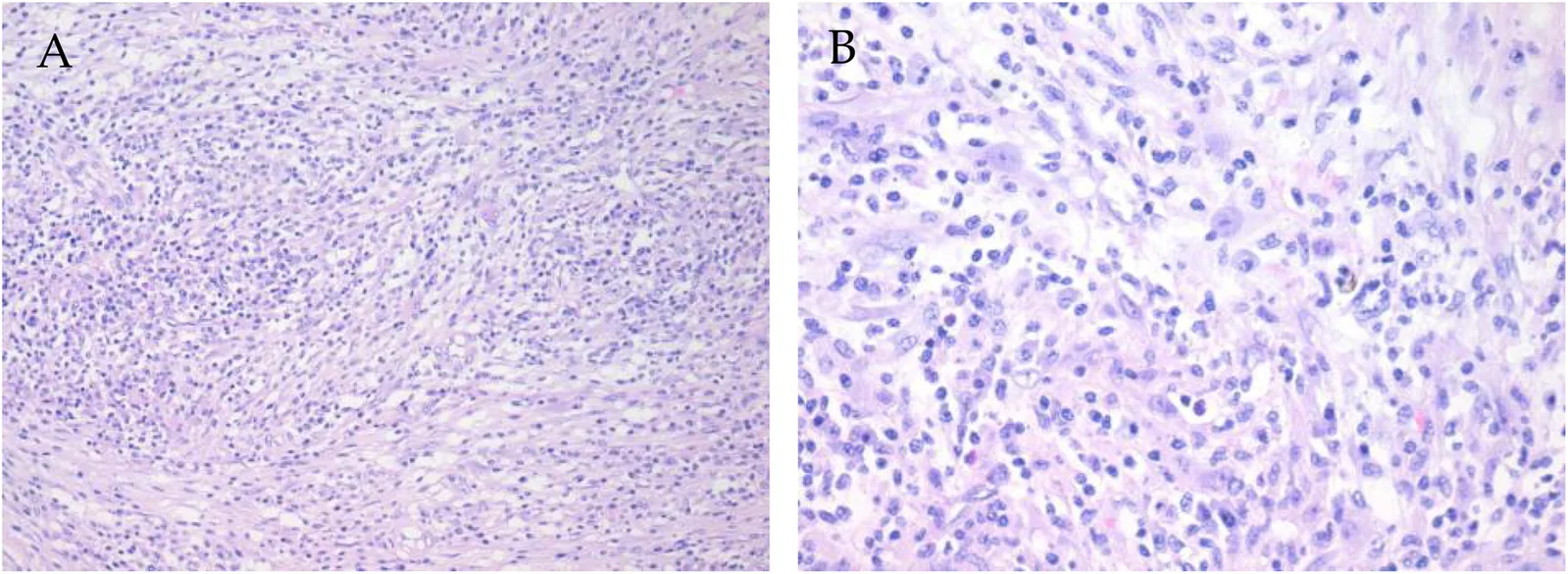
The postoperative pathological biopsy of HIMT (H&E staining).

#### Case Ⅱ

A 12-year-old girl presented with recurrent fever and abdominal pain. Laboratory studies revealed an elevated CRP level (23.6 mg/L), an erythrocyte sedimentation rate (ESR) of 55 mm/h, and a CD4+/CD8+ T-cell ratio of 1.97. Serologic testing demonstrated positive cytomegalovirus IgG (117.0 U/mL) and elevated Epstein–Barr virus (EBV) antibodies (EBV nuclear antigen IgG >600.0 U/mL; viral capsid antigen IgG 38.5 U/mL), consistent with past infection. All other serologic and microbiologic evaluations were negative. Abdominal ultrasonography revealed abnormal hepatic echogenicity in the right lobe and enlarged hilar lymph nodes, raising suspicion for a hepatic abscess (Figure 1B). Abdominal CT revealed a subcapsular lesion in hepatic segment V, with associated biliary tract changes in the right lobe, suggestive of a hepatic abscess complicated by cholangitis (Figures 2B,C). Empiric antibiotic therapy was initiated. After 4 days of treatment, inflammatory markers normalized, and the patient became afebrile and asymptomatic, prompting discharge.

One week postdischarge, she returned with recurrent fever and abdominal pain. Abdominal MRI again demonstrated findings suggestive of a hepatic abscess (Figures 3F–J). Due to persistent symptoms despite prior therapy, she underwent laparoscopic exploration with partial excisional biopsy. Intraoperative examination revealed adhesions between hepatic segment V and the anterior abdominal wall. The hepatic lesion lacked clear demarcation from the surrounding parenchyma, and the liver capsule exhibited multiple white, punctate, miliary exudates. Marked right lobe enlargement was observed, with the gallbladder adherent to the lesion and mildly distended. Initial histopathologic evaluation suggested a possible HIMT (Figures 6A,B). Immunohistochemical staining was negative for *ALK* and *NTRK*, and positive for *SMA*. Partial expression of IgG and IgG4 was observed, with an IgG4/IgG ratio <30%, a value below the established diagnostic cutoff for IgG4-related disease. Masson trichrome staining demonstrated prominent fibrous proliferation. *In situ* hybridization for EBV-encoded RNAs (EBERs) was negative. Metagenomic next-generation sequencing (mNGS) of tissue identified no pathogenic organisms, and targeted next-generation sequencing (NGS) using a DNA- and RNA-based hybrid-capture assay (Sarcoma PanScan^TM^) revealed no oncogenic mutations associated with soft tissue sarcomas.

**Figure 6:**
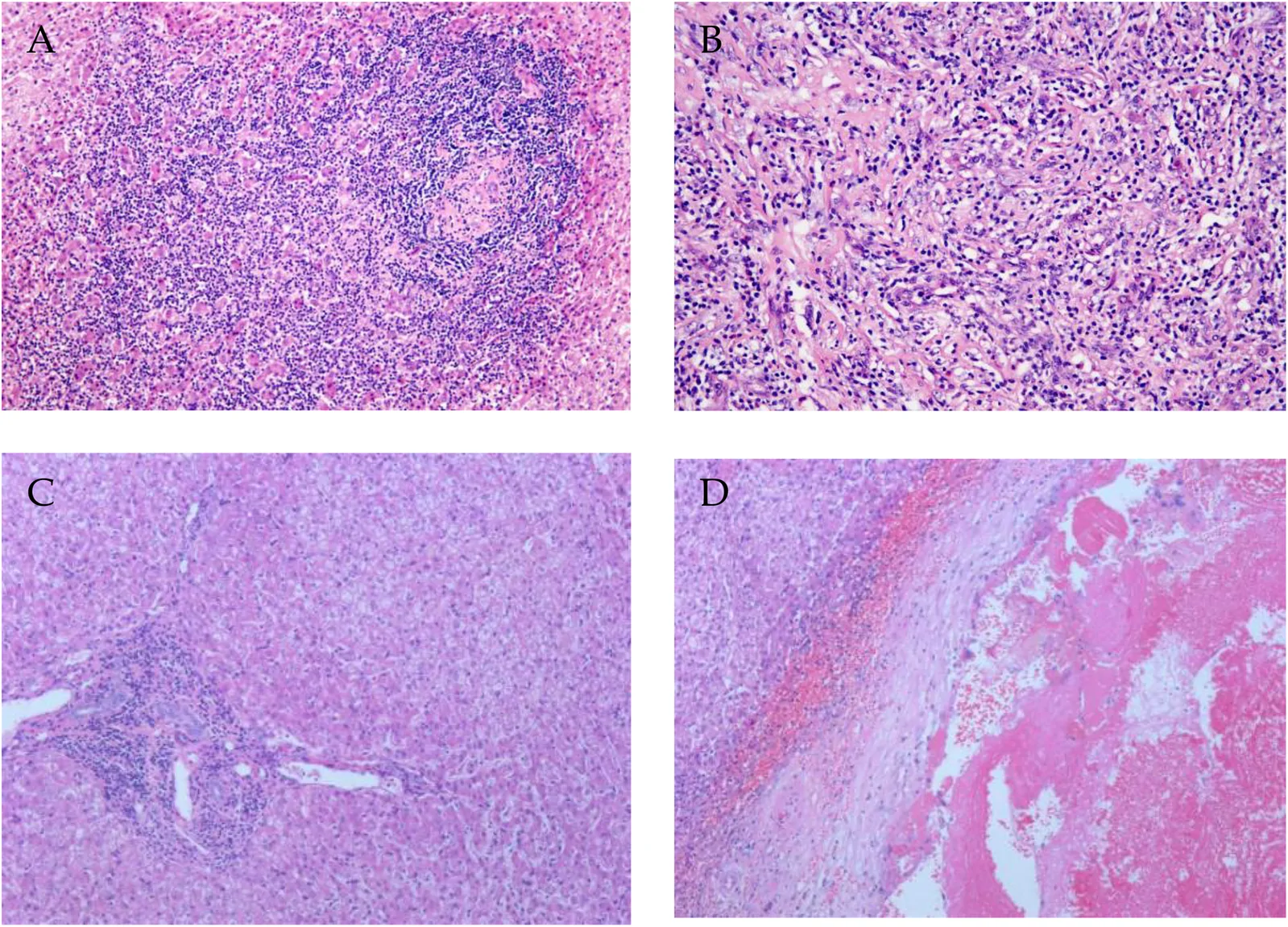
The postoperative pathological biopsy of HIPT (H&E staining).

Given the diagnostic uncertainty, a second, more extensive surgical resection was subsequently performed. Intraoperative findings revealed persistent miliary exudates on the hepatic capsule. The lesion in segment V was more clearly demarcated than during the initial procedure, with minimal surface exudate. Following multidisciplinary pathologic review, the histologic features were ultimately consistent with a HIPT (Figures 6C,D). Repeat immunohistochemistry confirmed ALK and IgG4 negativity, with positive staining for *SMA, HepPar-1, cytokeratin 7 (CK7), CK8/18, β-catenin, CD45, CD68, CD38*, and *CD235a.* The presence of HepPar-1, CK7, CK8/18, and *β*-catenin was seen as indicating trapped hepatocytes and bile duct epithelium, while CD45, CD68, CD38, and CD235a marked the mixed inflammatory and hematopoietic cell infiltrate. Special stains again highlighted extensive fibrous tissue proliferation. NGS analysis revealed no detectable mutations in a panel of genes, including *ALK*, *ROS1*, *KRAS*, *NRAS*, and *BRAF*. After surgery, the patient was given cefoperazone-sulbactam for antibacterial prevention and a small dose of prednisone for corticosteroid treatment. Positron emission tomography–computed tomography (PET-CT) at 6 months postoperatively demonstrated no evidence of residual disease or metastasis. At 12-month follow-up, the patient remained asymptomatic with no recurrence of fever or abdominal pain.

## Discussion

Pediatric HIMT and HIPT are extremely rare masses, and their differential diagnosis remains a significant challenge. We compiled 18 HIMT and 11 HIPT pediatric cases over the past 15 years, including two index cases from our institutions reported herein, and analyzed their clinical characteristics. The following discussion will address their demographic characteristics, etiology, clinical manifestations, imaging findings, treatment modalities, and pathology.

According to previous literature, the typical age of onset for IMT is childhood and adolescence, with a male-to-female ratio of 2:1 (11); by contrast, IPT is more commonly observed in adult female patients (12). No significant gender bias was identified in pediatric HIMT or HIPT in the current research. The age of onset varied between the two groups: 12 out of 18 pediatric HIMT cases were younger than 6 years, while most pediatric HIPT cases happened during adolescence, with 8 out of 11 occurring between 10 and 15 years (Table 1).

**Table 1:** Baseline characteristics of HIMT and HIPT.

Features	IMT(*n* = 18)	IPT(*n* = 11)
Clinical features		
Age(years)		
0∼3	7	1
4∼6	5	0
7∼9	3	2
10∼12	1	3
13∼15	2	5
Sex		
Male	10	6
Female	8	5
Size		
＜5cm	5	3
≥5cm	8	7
Unknown	5	1
Location		
Right lobe	7	6
Hepatic hilum	2	2
Left lobe	7	1
Entrance of the portal vein into the liver	0	1
caudate lobe	0	1
Unknown	2	0

Data are numbers (%).

Potential etiologies of IMT include inflammation, trauma, autoimmune diseases, surgery-related factors, and infection (13, 14), while possible etiologies of IPT include trauma, autoimmune diseases, cholangitis, and viral infection (15). The previously reported EBV-associated cases have been classified as inflammatory pseudotumor-like follicular/fibroblastic dendritic cell sarcoma, a distinct neoplastic entity that should not be conflated with conventional HIPT (16, 17). In Case II, the patient had serologic evidence of past EBV exposure (EBV-IgG positivity), but EBER *in situ* hybridization of the lesion was negative. Therefore, the present case does not provide direct evidence supporting an aetiologic role for EBV in HIPT. Unfortunately, few of the pediatric hepatic IMT and IPT cases we collected (Tables 2, 3) described etiologies or predisposing factors. Thus, further research is needed to ascertain the true etiologies of HIPT and HIMT.

**Table 2:** Clinical and pathological data of HIMT.

Case	Age（y）/sex	Presentation	Size (cm)	Hepatic lobe	Liver abscess	ALK-1 expression	Treatment/efficacy	Years of follow-up	Recurrence	Survival
1 (23)	4/F	vomiting, fecal acholia, jaundice, and itching of the upper limbs	-	Right lobe	No	-	CO/ ineffective；CE/ effective	0.5	No	Yes
2 (24)	3/F	post wilms tumor surveillance	-	-	No	No	CE/ effective	9	No	Yes
3	15/M	night sweats, fever and tiredness	-	-	No	No	CE/ effective	9	No	Yes
4 (26)	10/F	fever and abdominal pain	5.6	Hepatic hilum	No	No	CO，NAD/ effective	2	No	Yes
5 (27)	0/M	abdominal distension，ascites	7	Left lobe	Yes	No	CO，CE/ effective	7	No	Yes
6 (28)	3/F	fever	-	Right lobe	Yes	-	AN/ effective	0.5	No	Yes
7 (29)	6/M	abdominal pain	4.5	Right lobe	-	No	CE/ effective	2	No	Yes
8	1/M	abdominal distension, fever, vomiting	8	Left lobe	-	Yes	CE/ effective	10	No	Yes
9	4/F	abdominal pain	7	Left lobe	-	Yes	CE/ effective	1	No	Yes
10 (30)	3/M	jaundice, choluria, fecal acholia, fever and abdominal pain	8.0	Hepatic hilum	No	No	CE，LT/ effective	3	No	Yes
11 (31)	0.4/M	hepatomegaly	10.4	Right lobe	No	Yes	CE/ effective	1	No	Yes
12 (32)	4/M	Abdominal pain	4	Right lobe	-	No	CE/ effective	5	No	Yes
13 (33)	1/M	hepatomegaly, anemia	5.0	Left lobe	No	-	CE/ effective	1	No	Yes
14 (34)	14/M	Thoracic wall tumor，anemia	-	Right lobe	No	No	AN/ effective	6	No	Yes
15 (35)	9/F	Abdominal pain，fever	3.5	Left lobe	No	No	CE, ATT/ effective	3	No	Yes
16 (36)	8/M	Jaundice; pain in the right hypochondrium， acholic stools， hyperchromic urine	35	Left lobe	No	-	ID,CT/ineffective TT/ongoing	-	-	Yes
17	4/F	Vomiting, diarrhea, jaundice, clay-colored feces, itching	4.8	Right lobe	No	-	CO,CE/effective	-	-	Yes
18^a^	8/F	chronic abdominal pain, anemia	4.9	Left lobe	No	Yes	CE/effective	1	No	Yes

AN, antibiotherapy; ATT, empiric anti-tubercular therapy; CE, complete resection; CO, Corticosteroid; IE, interventional embolisation; ID, interventional drainage; IM, immunosuppressant; LT, liver transplantation, NAD: nonsteroidal anti-inflammatory drug; CT, chemotherapy; TT, targeted therapy; y, year; -, not available.

a The cases of our center (Case).

**Table 3:** Clinical and pathological data of HIPT.

Case	Age（y）/sex	Presentation	Size (cm)	Hepatic lobe	Liver abscess	ALK-1 expression	Treatment/efficacy	Years of follow-up	Recurrence	Survival
19 (37)	3/M	fever，anemia	7.6	Right lobe	No	-	CO, IM/ effective	0.6	No	Yes
20 (38)	12/F	fever，anemia	4.4	Hepatic hilum	No	-	AN，CO，NSAID/ ineffective; CE/ effective	4	No	Yes
21	13/F	fever，anemia	5.4	Hepatic hilum	Yes	-	CE/ effective	1	No	Yes
22 (39)	14/M	fever, jaundice	9.5	Right lobe	Yes	-	CO, ATT, AN, ID/ effective	0.3	No	Yes
23 (19)	8/M	hepatic mass, fever	6.6	Right lobe	No	No	CE/ effective	0.3	No	Yes
24 (40)	9/M	fever, abdominal pain	-	Right lobe	No	-	AN/ effective	0.3	No	Yes
25 (50)	14/M	Fever, chills, and anorexia	5.9	Left lobe	No	-	CE/ effective	1	No	Yes
26 （^41^）	15/F	tiredness, fever, and weight loss	4.9	Entrance of the portal vein into the liver	-	No	CE/ effective	6	No	Yes
27	14/F	itchy urticarial exanthema on the face	5.0	caudate lobe	-	-	CE/ effective	12	No	Yes
28 (42)	11/M	abdominal pain, anorexia, weight loss and fever	7.4	Right lobe	-	-	NAD/ effective	1	No	Yes
29^a^	12/F	fever, abdominal pain	2.3	Right lobe	Yes	No	AN/ineffective; CE, CO/effective	1	No	Yes

AN, antibiotherapy; ATT, empiric anti-tubercular therapy; CE, complete resection; CO, Corticosteroid; IE, interventional embolisation; ID, interventional drainage; IM, immunosuppressant; LT, liver transplantation; NAD: nonsteroidal anti-inflammatory drug; y: year; -, not available.

a The cases of our center (Case).

Current literature generally agrees that the initial symptoms of HIMT and HIPT are similar, primarily including fever, abdominal pain, anemia, jaundice, and abdominal distension (8, 18, 19). Case I presented with chronic abdominal pain without systemic infectious symptoms, while case II exhibited recurrent fever and abdominal pain with prominent systemic inflammatory responses, accompanied by an abnormal CD4+/CD8⁺T-cell ratio. Our descriptive analysis indicated that the initial symptoms of HIMT are relatively diverse, with fever or abdominal pain being predominant, whereas almost all HIPT patients present with fever, and a considerable proportion have abdominal pain or anemia (Tables 2, 3). Regarding lesion location, our review shows that HIMT occurs equally in the left and right hepatic lobes (7/16 in each), while HIPT mainly occurs in the right lobe (6/11) (Table 1).

Regarding the imaging features of IMT, recent sonographic studies have reported that ultrasound typically reveals a solid hypoechoic mass with irregular morphology, uneven internal echoes, and increased vascularity on color Doppler flow imaging. Tumor borders may be well-defined or ill-defined, and internal blood flow signals are often enriched (20, 21). The ultrasound findings in Case I were consistent with these reports. According to the literature, the CT appearance of HIMT often shows heterogeneous enhancement (47%) and peripheral enhancement (36%), whereas MRI predominantly shows hypointensity on T1-weighted images (77%) and hyperintensity on T2-weighted images (90%) (18). The imaging findings in Case I were similar to these commonly reported features; however, the value of CT and MRI in aiding differential diagnosis requires further investigation. Regarding the imaging features of IPT, a recent multimodal imaging study of HIPT reported that the misdiagnosis rate for individual ultrasound, CT, or MRI contrast-enhanced examinations exceeds 50% (22). At our center, based on CT and MRI findings, Case II was initially considered a liver abscess, also resulting in misdiagnosis. The same multimodal imaging study reported that HIPT most frequently appears as a hypoechoic lesion (83.5%) with irregular morphology (62.0%), heterogeneous echotexture (72.2%), and ill-defined margins (65.8%) on conventional ultrasound. Characteristic differences exist in the washout pattern between contrast-enhanced ultrasound (CEUS) and contrast-enhanced CT/MRI: HIPT demonstrates rapid washout on CEUS, whereas it exhibits delayed and persistent enhancement on CT/MRI (22). Therefore, multimodal imaging is highly valuable for diagnosing HIPT, and further investigation into the differing washout patterns between CEUS and contrast-enhanced CT/MRI is warranted. Overall, although most IMT and IPT patients reported in the literature have undergone ultrasound, CT, and MRI examinations, dedicated studies specifically investigating the imaging characteristics of these two diseases are scarce, and studies focusing on the pediatric population are even rarer. Hence, relying solely on imaging modalities to diagnose HIMT and HIPT is highly challenging and prone to misdiagnosis.

Regarding the treatment of pediatric HIMT and HIPT, there are currently no uniform treatment guidelines or expert consensus. Based on the individual cases we collected (Table 2), the mainstay treatment for HIMT was complete resection (14/18), with remaining treatments including corticosteroids, nonsteroidal anti-inflammatory drugs (NSAIDs), antibiotics, chemotherapy, and targeted therapy, all associated with favorable outcomes (23–36). According to our investigation (Table 3), treatment modalities for HIPT include surgical resection alone (5/11), corticosteroids or anti-inflammatory medication combined with surgery (2/11), immunosuppressants, and anti-inflammatory medication alone (17, 19, 37–42). Patients in case 19, 22, 24, and 28 did not undergo surgical treatment but ultimately achieved favorable outcomes, suggesting that treatment approaches for HIPT can be diverse. In Case II, the HIPT patient was initially misdiagnosed with a liver abscess and thus received antibiotics, which did not achieve sustained resolution, necessitating further investigation and surgical intervention. Only after repeated discussions among pathology experts was the mass definitively identified as HIPT. Therefore, complete surgical resection remains the cornerstone of treatment for HIMT when it can be achieved safely. However, for unresectable, recurrent, or anatomically complex lesions, systemic therapy—particularly targeted therapy in tumors harboring *ALK, ROS1,* or *NTRK* rearrangements—may be beneficial, and neoadjuvant treatment may enable a less extensive, organ-preserving procedure (9, 10, 21, 43). In contrast, because HIPT is an inflammatory, non-neoplastic lesion, the appropriate treatment should be selected according to the patient's actual condition: if the lesion is small and symptoms are mild, a trial of medical therapy such as NSAIDs or corticosteroids may be prioritized; however, if the lesion is extensive, if symptoms persist or recur despite treatment, or if malignancy cannot be confidently excluded, surgical resection or a more extensive biopsy should be considered.

Regarding histopathology, the literature reports that IMT pathological tissue microscopically consists of fascicular or haphazardly arranged spindle-shaped fibroblasts or myofibroblasts embedded in a fibrous stroma containing a variable myxoid matrix, interspersed with lymphocytes, granulocytes, and plasma cells. In HIMT, the bile ducts surrounding the tumor lack the inflammatory changes typical of sclerosing cholangitis (44, 45). Other literature proposes three basic histological patterns for IMT based on the characteristics of fibroblasts, myofibroblasts, inflammatory cells, and cytoplasm: myxoid/vascular, compact spindle cell, and hypocellular fibrous. The myxoid/vascular pattern comprises loosely arranged, variably shaped myofibroblasts within a highly vascularized stroma, accompanied by abundant infiltrates of plasma cells, eosinophils, and lymphocytes. The compact spindle cell pattern is characterized by closely packed fascicles of spindle cells with varying degrees of myxoid and collagenous stroma and inflammatory infiltration. The hypocellular fibrous pattern features a hyalinized collagenous stroma with low spindle cell density and relatively sparse inflammatory infiltration, resembling a scar or desmoid fibromatosis (46, 47). Immunohistochemically, fibroblastic/myofibroblastic spindle cells generally express *SMA*, and most express desmin and calponin. Clonal translocations involving the *ALK* receptor tyrosine kinase gene occur in over 60% of cases, and even tumors negative for *ALK* rearrangement may harbor other gene rearrangements, such as *ROS1* (44). Therefore, detecting *ALK* or *ROS1* rearrangement may aid in diagnosing IMT. Case I exhibited histopathological morphology consistent with the myxoid/vascular pattern, and immunohistochemistry was positive for *ALK*, aligning with literature reports. Currently, most case reports do not provide detailed immunohistochemistry information; some even omit this component entirely, hindering the compilation and study of HIMT-related case data—an issue that requires future improvement. Regarding HIPT, its microscopic appearance closely resembles that of HIMT, both featuring infiltration of spindle-shaped fibroblasts or myofibroblasts and inflammatory cells. The inflammatory cells consist predominantly of plasma cells, macrophages, and lymphocytes, with fibrotic or necrotic areas surrounding the lesion but lacking cellular atypia (48, 49). According to the literature, HIPT can be pathologically divided into fibrohistiocytic and lymphoplasmacytic types. The former is characterized by xanthogranulomatous inflammation with infiltrating multinucleated giant cells and neutrophils, expresses SMA, and often presents as a mass lesion in the peripheral hepatic parenchyma. The latter manifests as diffuse infiltration of lymphocytes and plasma cells, prominent eosinophilic infiltration, and abundant IgG4-positive plasma cells, typically located around the hepatic hilum (50). Case II showed histopathological features of the lymphoplasmacytic type, and immunohistochemistry was negative for ALK expression, consistent with literature reports. Evidently, the pathological manifestations of HIMT and HIPT are also extremely similar. Therefore, some experts propose that judicious use of immunohistochemistry and molecular testing—such as for *ALK*, *ROS1*, and *NTRK*—can serve as crucial ancillary evidence to differentiate IMT from IPT (51, 52).

Drawing upon a comprehensive array of information encompassing clinical manifestations, radiological characteristics, pathological findings, and molecular testing outcomes, we have devised a proposed diagnostic framework to facilitate differential diagnosis (Figure 7).

**Figure 7:**
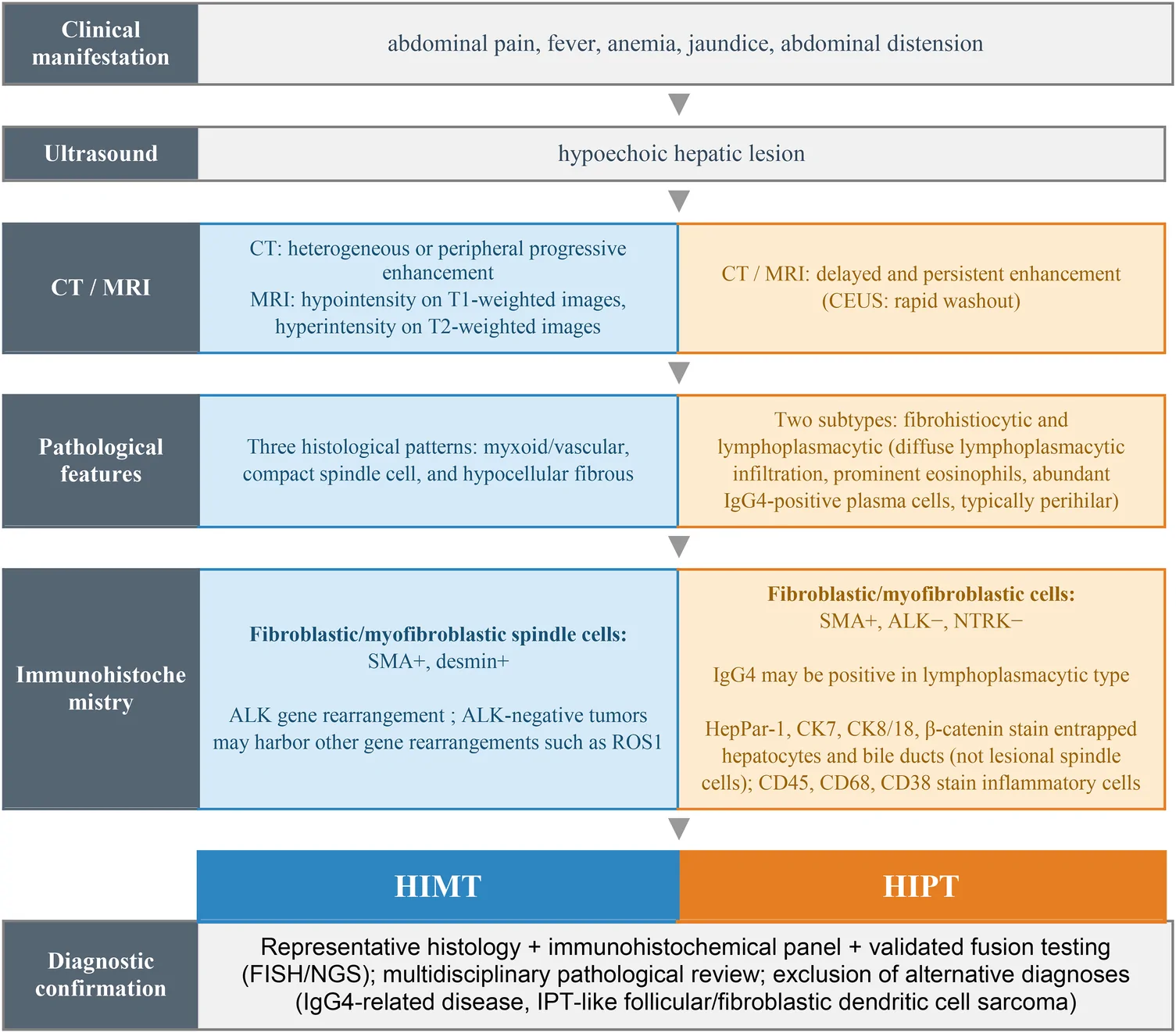
Proposed diagnostic framework for the differential diagnosis of HIMT and HIPT.

## Limitations

The small institutional sample and the inherent selection and publication biases of literature-based reviews preclude definitive conclusions. Historical heterogeneity in terminology and classification, incomplete molecular data in older reports, and the potential for duplicate publication further limit data interpretation. Variability in follow-up duration across included studies restricts outcome assessment, and the proposed diagnostic framework lacks external validation. These findings should therefore be interpreted as exploratory.

## Conclusions

In summary, as HIMT is a true neoplasm with potential for recurrence, distant metastasis, and local invasion, surgical excision remains the mainstay of treatment. In contrast, HIPT can sometimes be successfully managed with medical therapies—including antibiotics, anti-inflammatory drugs and corticosteroids—thus avoiding surgical trauma. However, HIMT and HIPT are highly similar, making differentiation considerably challenging. Consequently, accurate distinction between these two entities is critical. Multicenter clinical studies are essential to further elucidate the differences between HIPT and HIMT and to explore their underlying pathogenesis.

## Data Availability

The original contributions presented in the study are included in the article/Supplementary Material, further inquiries can be directed to the corresponding author.

## References

[1] Umiker WO Iverson L. Postinflammatory tumors of the lung; report of four cases simulating xanthoma, fibroma, or plasma cell tumor. J Thoracic Surg. (1954) 28(1):55–63. 10.1016/S0096-5588(20)30758-3 13175281

[2] Brown G Shaw DG. Inflammatory pseudotumours in children: cT and ultrasound appearances with histopathological correlation. Clin Radiol. (1995) 50(11):782–6. 10.1016/S0009-9260(05)83220-9 7489630

[3] Coffin CM Fletcher CDM Inflammatory myofibroblastic tumor. In: Fletcher CDM Unni KK Mertens F, editors. World Health Organization Classification of Tumours: Pathology and Genetics of Tumours of Soft Tissue and Bone. 3rd ed. Lyon: IARC Press; 2002. p. 91–3.

[4] Dehner LP. Inflammatory myofibroblastic tumor: the continued definition of one type of so-called inflammatory pseudotumor. Am J Surg Pathol. (2004) 28(12):1652–4. 10.1097/00000478-200412000-00016 15577687

[5] Fabre D Fadel E Singhal S De Montpreville V Mussot S Mercier O. Complete resection of pulmonary inflammatory pseudotumors has excellent long-term prognosis. J Thorac Cardiovasc Surg. (2009) 137(2):435–40. 10.1016/j.jtcvs.2008.07.009 19185166

[6] Fornell-Pérez R Santana-Montesdeoca JM García-Villar C MC C-G. Two types of presentation of pulmonary inflammatory pseudotumors. Arch Bronconeumol. (2012) 48(8):296–9. 10.1016/j.arbr.2012.05.005 22075403

[7] Mattei P Barnaby K. Rapid regression of duodenal inflammatory myofibroblastic tumor after intravenous ketorolac: case report and review of the literature. J Pediatr Surg. (2008) 43(6):1196–9. 10.1016/j.jpedsurg.2008.01.012 18558206

[8] Fu GX Xu CC Yao NF Gu JZ Jiang HL Han XF. Inflammatory myofibroblastic tumor: a demographic, clinical and therapeutic study of 92 cases. Math Biosci Eng. (2019) 16(6):6794–804. 10.3934/mbe.2019339 31698588

[9] Pire A Orbach D Galmiche L Berrebi D Irtan S Boudjemaa S. Clinical, pathologic, and molecular features of inflammatory myofibroblastic tumors in children and adolescents. Pediatr Blood Cancer. (2022) 69(5):e29460. 10.1002/pbc.29460 34854544

[10] Casanova M Brennan B Alaggio R Kelsey A Orbach D Van Noesel MM. Inflammatory myofibroblastic tumor: the experience of the European pediatric soft tissue sarcoma study group (EpSSG). Eur J Cancer. (2020) 127:123–9. 10.1016/j.ejca.2019.12.021 32007712

[11] Mehta B Mascarenhas L Zhou S Wang L Venkatramani R. Inflammatory myofibroblastic tumors in childhood. Pediatr Hematol Oncol. (2013) 30(7):640–5. 10.3109/08880018.2013.816810 23988029

[12] Goldsmith PJ Loganathan A Jacob M Ahmad N Toogood GJ Lodge JPA. Inflammatory pseudotumours of the liver: a spectrum of presentation and management options. Eur J Surg Oncol. (2009) 35(12):1295–8. 10.1016/j.ejso.2009.04.003 19515527

[13] Xiang J Liu X Wu S Lv Y Wang H. Multiple inflammatory myofibroblastic tumor of the duodenum: case report and literature review. J Gastrointest Surg. (2012) 16(7):1442–5. 10.1007/s11605-012-1883-9 22528570

[14] Tao YL Wang ZJ Han JG Wei P. Inflammatory myofibroblastic tumor successfully treated with chemotherapy and nonsteroidals: a case report. World J Gastroenterol. (2012) 18(47):7100–3. 10.3748/wjg.v18.i47.7100 23323014 PMC3531700

[15] Patnana M Sevrukov AB Elsayes KM Viswanathan C Lubner M Menias CO. Inflammatory pseudotumor: the great mimicker. Am J Roentgenol. (2012) 198(3):W217–27. 10.2214/AJR.11.7288 22358018

[16] Pagliuca F Ronchi A Auricchio A Lieto E Franco R. Inflammatory pseudotumor-like follicular/fibroblastic dendritic cell sarcoma: focus on immunohistochemical profile and association with epstein–barr virus. Infect Agents Cancer. (2022) 17(1):63. 10.1186/s13027-022-00474-8 PMC9789662 36566201

[17] Li H-L Liu H-P Guo GW-J Chen Z-H Zhou F-Q Liu P. Imaging findings of inflammatory pseudotumor-like follicular dendritic cell tumors of the liver: two case reports and literature review. World J Gastroenterol. (2019) 25(45):6693–703. 10.3748/wjg.v25.i45.6693 31832007 PMC6906205

[18] Tang L Lai ECH Cong W- Li A- Fu S- Pan Z- Inflammatory myofibroblastic tumor of the liver: a cohort study. World J Surg. (2010) 34(2):309–13. 10.1007/s00268-009-0330-x 20033408

[19] Al-Hussaini H Azouz H Abu-Zaid A. Hepatic inflammatory pseudotumor presenting in an 8-year-old boy: a case report and review of literature. World J Gastroenterol. (2015) 21(28):8730–8. 10.3748/wjg.v21.i28.8730 26229415 PMC4515854

[20] Inoue M Ohta T Shioya H Sato S Takahashi H Nakata N. Inflammatory myofibroblastic tumors of the breast with simultaneous intracranial, lung, and pancreas involvement: ultrasonographic findings and a review of the literature. J Med Ultrasound. (2001). 2018) 45(2):331–5. 10.1007/s10396-017-0829-y 29027063

[21] Guo F Cui Q Lei Y Xi H. Inflammatory myofibroblastic tumors in children: a single-center retrospective study of clinical features, management, and outcomes. Front Pediatr. (2025) 13:1667711. 10.3389/fped.2025.1667711 40980125 PMC12443843

[22] Chen K Luo M He Y Huang D Tang M Shi J. Clinical and multimodal imaging features of hepatic inflammatory pseudotumors: a two-center retrospective study. J Ultrasound Med. (2025) 44(4):691–701. 10.1002/jum.16629 39665519

[23] Napolitano M Lama B Ierardi AM Valle C Rossi E Minelli R. A case report of hepatic inflammatory myofibroblastic tumor in a pediatric patient: diagnostic challenges and management strategies. J Ultrasound. (2024) 29:229–36. 10.1007/s40477-024-00975-9 39688771 PMC13000088

[24] Couper MR Eldredge JA Kirby M Kirby C Moore D Hammond P. Paediatric gastrointestinal, hepatic and pancreatic inflammatory myofibroblastic tumours, A single centre experience. J Pediatr Gastroenterol Nutr. (2022) 74(2):253–7. 10.1097/MPG.0000000000003311 34596604

[25] Thavamani A Mandelia C Anderson PM Radhakrishnan K. Pediatric inflammatory myofibroblastic tumor of the liver: a rare cause of portal hypertension. ACG Case Rep J. (2019) 6(3):1–4. 10.14309/crj.0000000000000028 31620493 PMC6658019

[26] Kato H Mitani Y Goda T Ueno M Hayami S Tsujimoto H. Hepatic inflammatory myofibroblastic tumor detected in the fetal period that caused an oncologic emergency. Case Rep Oncol. (2020) 13(3):1513–9. 10.1159/000511052 PMC7841740 33564292

[27] Rodrigues S Ferreira F Dias S. Liver abscess complicated with an inflammatory myofibroblastic tumor. J Belgian Soc Radiol. (2022) 106(1):78. 10.5334/jbsr.2756 PMC9503893 36213372

[28] Wan YY Miao CL Liu SB Zhang T Luo CH. Epithelioid inflammatory myofibroblastic sarcoma with leukemoid reaction. J Coll Physicians Surg Pak. (2022) 32(9):1212–5. 10.29271/jcpsp.2022.09.1212 36089724

[29] Han Q Zhang Z He X Chen M Pang X Chen C. Primary inflammatory myofibroblastic tumour of the liver: a clinicopathological and genetic study including a subset with ETV6::NTRK3 fusion. Histopathology. (2023) 82(6):925–36. 10.1111/his.14881 36748182

[30] Costa CM Neto JS Benavidez MR Vincenzi R Roda K Oliveira CMV. Liver transplantation for hilar inflammatory myofibroblastic tumor: case report and review of the literature. Pediatr Transplant. (2023) 27(3):e14445. 10.1111/petr.14445 36435975

[31] Kim SH Cho YH Kim HY. Two cases of infantile intra-abdominal inflammatory myofibroblastic tumor. Pediatr Gastroenterol Hepatol Nutr. (2014) 17(2):116–20. 10.5223/pghn.2014.17.2.116 25061588 PMC4107219

[32] Xiao Y Zhou S Ma C Luo J Zhu H Tang F. Radiological and histopathological features of hepatic inflammatory myofibroblastic tumour: analysis of 10 cases. Clin Radiol. (2013) 68(11):1114–20. 10.1016/j.crad.2013.05.097 23876410

[33] Nagarajan S Jayabose S McBride W Prasadh I Tanjavur V Marvin MR. Inflammatory myofibroblastic tumor of the liver in children. J Pediatr Gastroenterol Nutr. (2013) 57(3):277–80. 10.1097/MPG.0b013e31829e0b3b 23974058

[34] Fragoso AC Eloy C Estevão-Costa J Campos M Farinha N Lopes JM. Abdominal inflammatory myofibroblastic tumor a clinicopathologic study with reappraisal of biologic behavior. J Pediatr Surg. (2011) 46(11):2076–82. 10.1016/j.jpedsurg.2011.07.009 22075336

[35] Manolaki N Vaos G Zavras N Sbokou D Michael C Syriopoulou V. Inflammatory myofibroblastic tumor of the liver due to mycobacterium tuberculosis in an immunocompetent girl. Pediatr Surg Int. (2009) 25(5):451–4. 10.1007/s00383-009-2361-7 19396450

[36] Pierucci UM Paraboschi I Ardenghi C Viglio C Selvaggio GGO Lanfranchi G. Efficacy of core biopsies for diagnosing inflammatory myofibroblastic tumors in pediatric patients: case series from a single tertiary referral center. Transl Pediatr. (2024) 13(10):1799–809. 10.21037/tp-24-239 39524388 PMC11543115

[37] Wan Q Xu Z Liu X Wu Z Zhong Q Wu C. A case report of IgG4-related hepatic inflammatory pseudotumor in a 3-year old boy. Front Immunol. (2024) 15:1376276. 10.3389/fimmu.2024.1376276 38745658 PMC11091244

[38] Wiegering VA Klein I Wirth C Dick A Meyer T Schlegel PG. Anemia and B symptoms as leading symptoms for a hepatic inflammatory pseudotumor–2 case reports. Klin Padiatr. (2015) 227(6-7):363–5. 10.1055/s-0034-1395693 26038970

[39] Thanage R Jain S Sonthalia N Udgirkar S Chandnani S Contractor Q. An enigmatic liver mass in a child. Euroasian J Hepatogastroenterol. (2019) 9(2):104–7. 10.5005/jp-journals-10018-1307 32117700 PMC7047306

[40] Fernandes T Viamonte B Cunha R Trindade E Jesus JP. Peripheral inflammatory pseudotumor of the liver and extensive thrombosis of the portal venous system in a child. Pediatr Radiol. (2013) 43(8):1041–4. 10.1007/s00247-013-2655-8 23455309

[41] Rack A Belohradsky BH Grantzow R Schweinitz D Pfluger T Kammer B. Inflammatory pseudotumor (IPT)-surgical cure of an inflammatory syndrome. Eur J Pediatr. (2016) 175(7):903–8. 10.1007/s00431-016-2718-6 27073061

[42] Barros D Gomes F Osório A Oliveira da Silva P. Liver inflammatory pseudotumor in children: a case report. Pediatr Neonatol. (2021) 62(3):339–40. 10.1016/j.pedneo.2021.01.009 33612422

[43] Raitio A Losty PD. Treatment and outcomes in pediatric inflammatory myofibroblastic tumors - A systematic review of published studies. Eur J Surg Oncol. (2024) 50(7):108388. 10.1016/j.ejso.2024.108388 38713995

[44] Yamamoto H Yamaguchi H Aishima S Oda Y Kohashi K Oshiro Y. Inflammatory myofibroblastic tumor versus IgG4-related sclerosing disease and inflammatory pseudotumor: a comparative clinicopathologic study. Am J Surg Pathol. (2009) 33(9):1330–40. 10.1097/PAS.0b013e3181a5a207 19718789

[45] Buccoliero AM Ghionzoli M Castiglione F Paglierani M Baroni G Messineo A. Inflammatory myofibroblastic tumor: clinical, morphological, immunohistochemical and molecular features of a pediatric case. Pathol Res Pract. (2014) 210(12):1152–5. 10.1016/j.prp.2014.03.011 25149504

[46] Zhang Y Liu J. Inflammatory myofibroblastic tumor of the thyroid gland. Front Endocrinol (Lausanne). (2023) 14:1156117. 10.3389/fendo.2023.1156117 37255972 PMC10225677

[47] Sbaraglia M Bellan E Tos D P A. The 2020 WHO classification of soft tissue tumours: news and perspectives. Pathologica. (2021) 113(2):70–84. 10.32074/1591-951X-213 33179614 PMC8167394

[48] Kim SR Kim SK Koma YI Sasaki M Asai A Nishikawa H. Hepatic inflammatory pseudotumor-focusing on its heterogeneity. Diagnostics (Basel, Switzerland). (2023) 13(17). 10.3390/diagnostics13172857 PMC10486865 37685395

[49] Yang X Zhu J Biskup E Cai F Li A. Inflammatory pseudotumors of the liver: experience of 114 cases. Tumour Biol. (2015) 36(7):5143–8. 10.1007/s13277-015-3167-y 25663462

[50] Milias K Madhavan KK Bellamy C Garden OJ Parks RW. Inflammatory pseudotumors of the liver: experience of a specialist surgical unit. J Gastroenterol Hepatol. (2009) 24(9):1562–6. 10.1111/j.1440-1746.2009.05951.x 19744000

[51] Arora KS Anderson MA Neyaz A Yilmaz O Pankaj A Ferrone CR. Fibrohistiocytic variant of hepatic pseudotumor: an antibiotic responsive tumefactive lesion. Am J Surg Pathol. (2021) 45(10):1314–23. 10.1097/PAS.0000000000001767 34334689

[52] Taylor MS Chougule A MacLeay AR Kurzawa P Chebib I Le L. Morphologic overlap between inflammatory myofibroblastic tumor and IgG4-related disease: lessons from next-generation sequencing. Am J Surg Pathol. (2019) 43(3):314–24. 10.1097/PAS.0000000000001167 30451733 PMC6637760

